# American Health Primer—Brain Work and Overwork

**Published:** 1880-02

**Authors:** 


AMERICAN HEALTH PRIMER—BRAIN WORK AND OVERWORK. By Dr. H. C. Wood, Clinical Professor of Nervous Diseases in the University of Pennsylvania, etc., etc. PRESLY BLAKISTON, Publisher: Philadelphia. 1880.

